# Effect of dendrimeric composition on the removal of pyrene from water

**DOI:** 10.1186/s40064-015-1295-x

**Published:** 2015-09-17

**Authors:** Rebecca M. Triano, Michele L. Paccagnini, Amy M. Balija

**Affiliations:** Department of Chemistry, Fordham University, 441 East Fordham Road, Bronx, NY 10458 USA

**Keywords:** Dendrimer, Pyrene, Polycyclic aromatic hydrocarbon, Water pollution, Branched systems

## Abstract

Several closely related branched structures related to the well-established benzyl ether dendrimers were tested as hosts for the removal of pyrene from water. Fluorescence spectroscopy based complexation studies showed that the first generation benzyl ether dendrimers removed on average 94 % of pyrene from a saturated aqueous solution after 30 min. By contrast, branched systems containing modified benzyl ether or cyclohexane functional groups removed between 0 and 75 % of pyrene at the same time point while systems with benzyl amine resulted in a 77 % decrease in pyrene. This selectivity eroded over two days after which time all dendrimeric types removed pyrene to approximately the same extent. Inclusion constants of 10^9^–10^11^ M^−1^ were calculated for the branched systems studied.

## Background

Dendrimers are highly structured macromolecules composed of a central core connected to two or more repetitive branching units. The composition and molecular weight of these macromolecules are known precisely and can be controlled through synthetic transformations. Layers of monomers, or generations, are added by sequential synthetic iterations from the periphery or core of the dendrimer. The highly branched architecture makes dendrimers attractive for various applications including drug delivery, catalysis, light harvesting, and organic electronic devices (Astruc et al. 2010). Dendrimers possess interior voids which are ideal for entrapping small guests such as gas molecules, ions, and low molecular weight organic compounds (Zimmerman and Lawless 2001). This physical property has led to employing dendrimers in the field of environmental remediation.

Several investigations utilizing dendrimers as hosts for environmental pollutants complexation have been disclosed (Savage and Diallo 2005). Early examples focused on removing heavy metals such as copper, lead or chromium. Poly(amidoamine) (PAMAM) dendrimers removed Cu(II) from aqueous solutions (Diallo et al. 1999) and extracted Pb(IV) from contaminated soil samples (Xu and Zhao 2006). Simple modifications of the PAMAM periphery were shown to impact the type of metal ions removed. By preparing composites of TiO_2_ and PAMAM, Cu(II), Ni(II), and Cr(III) were eliminated from simulated wastewater (Barakat et al. 2013). Dendrimers have also been utilized to remove organic molecules. Ceramic filters impregnated with PAMAM and poly(ethyleneimine) (PEI) dendrimers were shown to extract pollutants such as trihalogen methanes, pesticides, and polycyclic aromatic hydrocarbons (PAHs) from water, the latter being a particularly persistent type of environmental toxin (Arkas et al. 2006). PAMAM and PEI dendrimers were also shown to solubilize phenanthrene, an example of a PAH, in a simulation of oil spill mitigation (Geitner et al. 2012). Other reports have established evidence for complex formation between PAH guests and dendrimers. For example, PAMAM dendrimers which were conjugated to fluorescent dye molecules effectively complexed phenanthrene through solution phase fluorescent resonant energy transfer (Lard et al. 2010) and the water-soluble dendrimers increased the aqueous solubility of pyrene through complex formation (Liu et al. 2000).

Many dendrimeric applications in environmental remediation employ commercially available macromolecules such as PEI or PAMAM. While serving as a proof of concept, these dendrimers are not optimized to complex particular environmental pollutants. There is much to learn regarding which dendrimeric structural features enhance small molecule complexation (Han and Gao 2011). The objective of the present work is to examine how subtle structural modifications impact the ability for dendrimers to remove small molecule organic pollutants, especially PAHs, from water. Towards this end, a series of closely related model branched systems and dendrimers were tested as hosts for pyrene. Preliminary studies were also done on anthracene and fluoranthene, alternative PAHs. The results reported herein will provide insight into the efficacy of the benzyl ether modified branching units for removing PAHs from an aqueous environment.

**Fig. 1:**
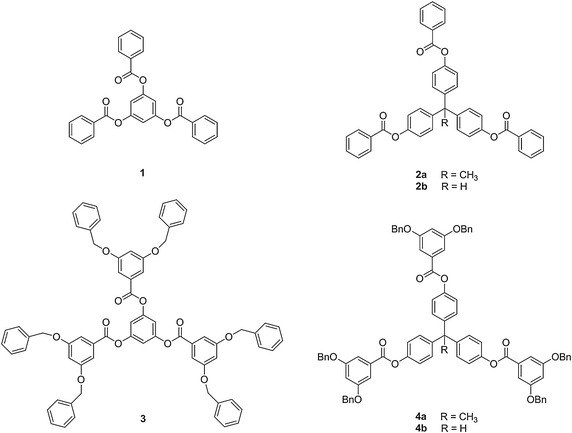
The structures of benzyl ether **1**–**4**

**Fig. 2:**
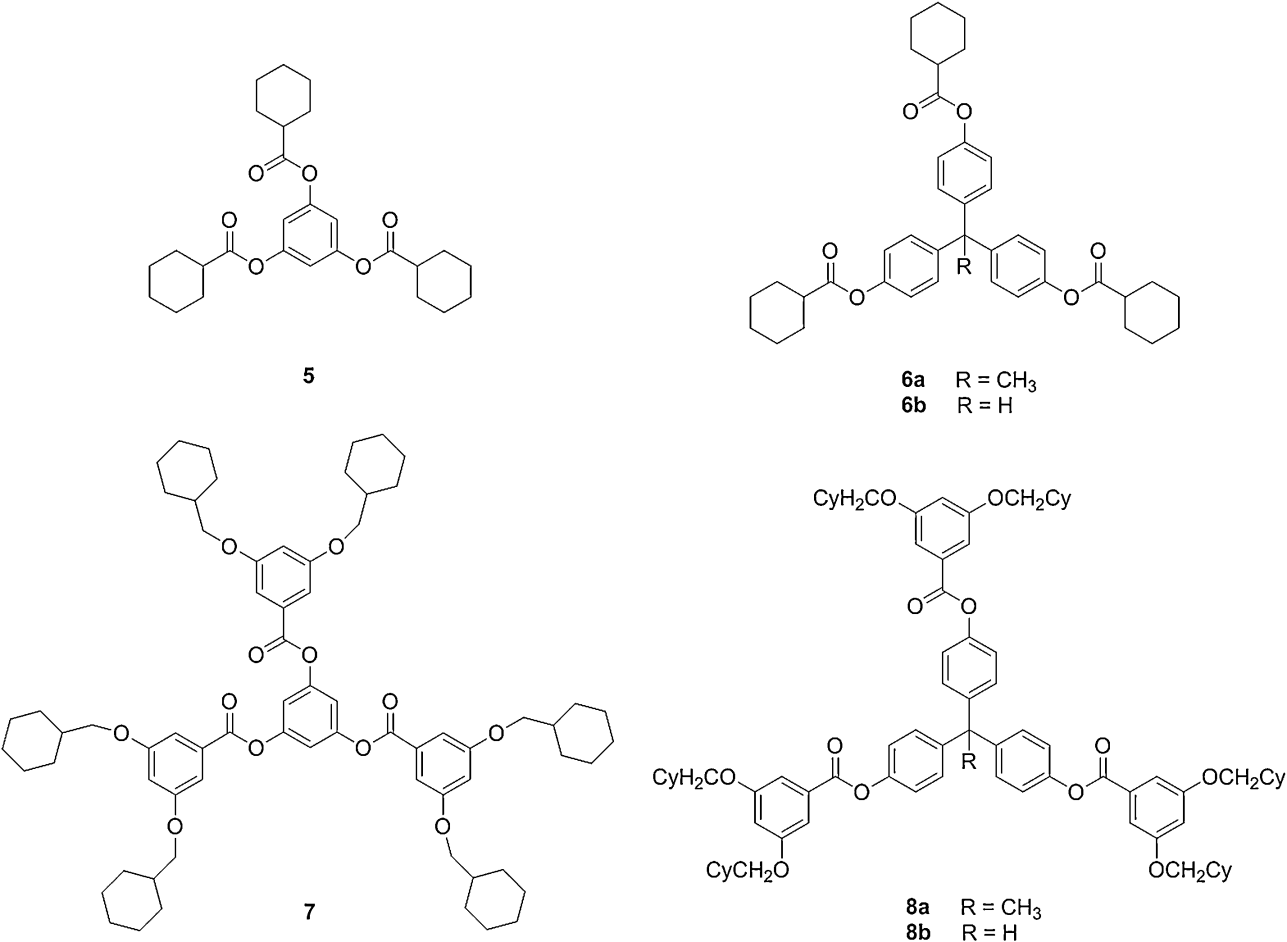
The structures of cyclohexane based **5**–**8**

**Fig. 3:**
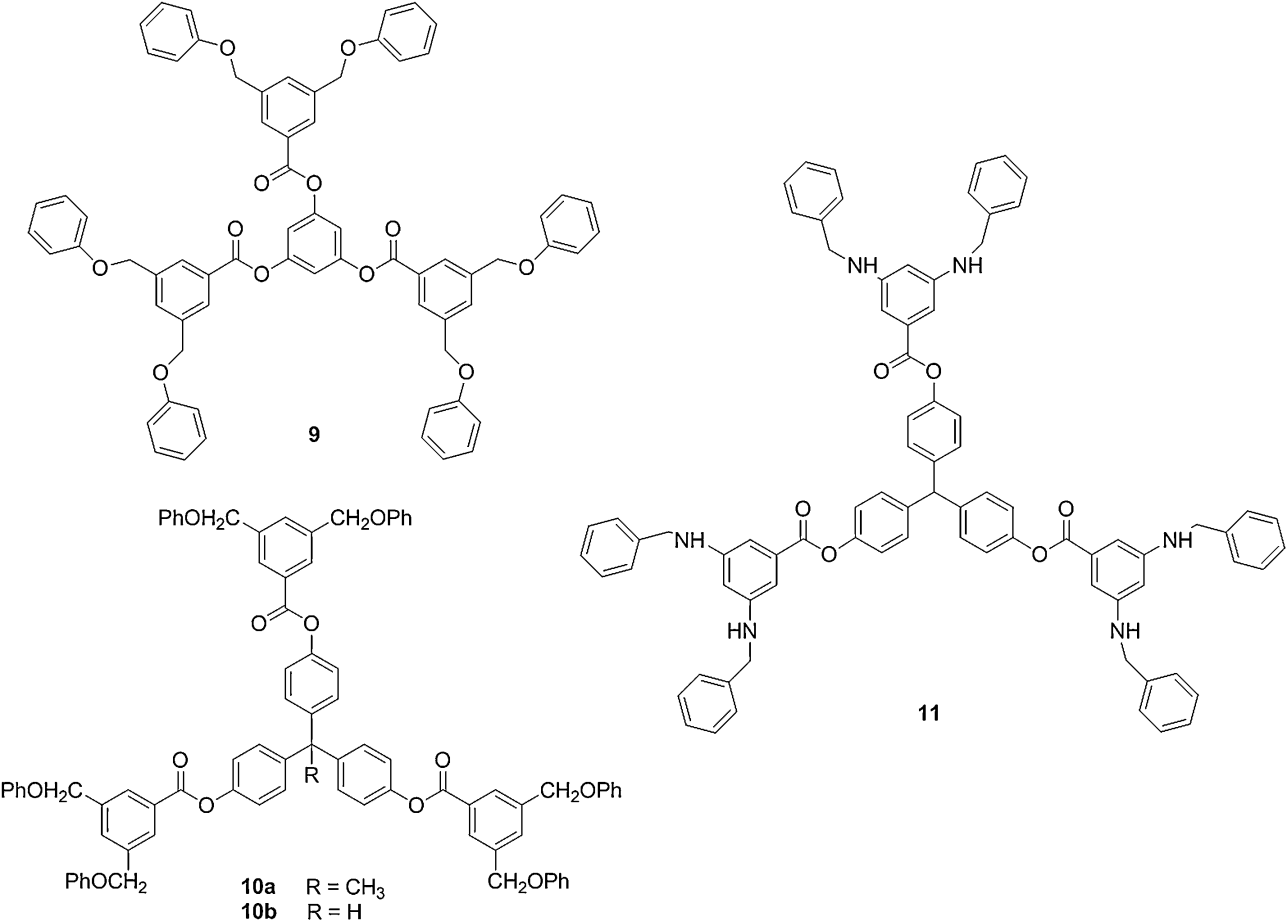
The structures of ‘reverse’ benzyl ethers **9**–**10** and benzyl amine **11**

Although several dendrimeric families have been reported, this research focuses on modifying the benzyl ether architecture, as shown in **1**–**4** (Fig. 1) (Hawker and Fréchet 1992). It is hypothesized that multiple benzene rings within these structures will favor complex formation with a PAH. The neutral framework of the benzyl ether design can be easily modified to prepare dendrimer families containing different functionalities and allow for a stepwise approach to examining how functional group placement impacts the physical properties of the resulting macromolecule (Hecht 2003). Because benzyl ether dendrimers have been employed for numerous applications, the results from this study can be applied to other purposes (Fréchet 2002; Sowinska and Urbanczyk-Lipowska 2014). Previous research has shown that smaller generation dendrimers provide similar results to their larger counterparts (Monaco et al. 2013). Thus, zero and first generation branched systems **1**–**11** (Figs. 1, 2, 3) were designed to provide comparable results to their larger analogs without the time consuming, multi-step syntheses needed to prepare the higher generation systems.

**Fig. 4:**
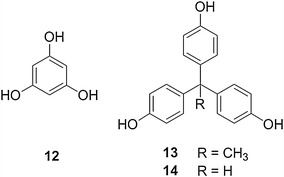
Cores utilized in the synthesis of **1**–**11**

Several objectives were considered in designing modified branched systems derived from **1**–**4**: (1) maintain the overall branching architecture of the benzyl ether system (2) design subtle structural changes in the dendrimer that could be readily synthesized, and (3) characterize the resulting systems using standard analytical techniques. Thus, a stepwise, informative approach to examining how composition influences dendrimer properties would be accomplished. Based upon the above factors, the following dendrimeric families were designed to be compared with benzyl ether **1**–**4**: systems **5**–**8** contained bulky cyclohexane groups along their periphery; ‘reverse’ benzyl ethers **9**–**10** were constitutional isomers of **3**–**4**; and benzyl amine **11** replaced the ether linkages with amine groups. Three different cores **12**–**14** were utilized to examine how small changes in the size and spatial configuration of the core impacted the overall dendrimer properties (Fig. 4). Thus, structure–activity relationships will be discovered which illustrate the effect of subtle architectural changes on the corresponding dendrimeric global properties.

## Results and discussion

**Fig. 5:**
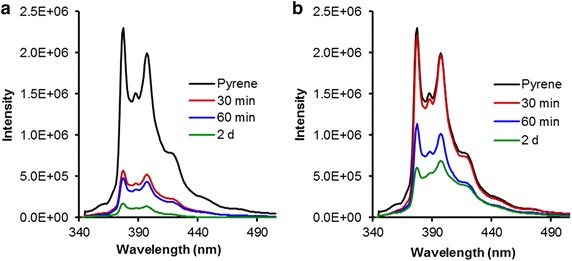
Pyrene fluorescence spectrum after exposure to **a** benzyl ether **2b** and **b** ‘reverse’ benzyl ether **10a**

The synthesis and characterization of systems **1**–**11** were previously reported (Triano et al. 2015; Monaco et al. 2013). Thin films containing the branched systems were exposed to saturated aqueous solutions of pyrene and aliquots of the aqueous solution were removed at specific time points and analyzed using fluorescence spectroscopy. Figure 5 shows the decrease in the fluorescence emission spectrum of an aqueous pyrene solution in the presence of **2b** and **10a** after 30 min, 60 min, and 2 days. This fluorescence decrease was attributed to one of three factors: (1) self-quenching of fluorescence due to pyrene aggregation in water, (2) a change in the polarity of the pyrene environment or (3) a decrease in the pyrene concentration in the aqueous phase (Bains et al. 2011). Self-quenching due to aggregation was eliminated by measuring the fluorescence spectrum of pyrene in water without the branched structure. No change in fluorescence intensity was observed during the 2 day period, indicating that spontaneous aggregation did not occur. Although the possibility exists that the solution polarity could change due to dendrimers leaching from the thin film into the solution phase, the low solubility of dendrimers such as **1**–**11** in water made it unlikely that any minor amount of leaching would significantly alter the dielectric constant of an aqueous solution (Liu et al. 1999). Thus, it was concluded that the changes in pyrene fluorescence observed and illustrated by Fig. 5 was due to removal of pyrene from the aqueous phase.

The spectral data (Fig. 5) was converted into tabular form to provide a facile way to interpret the data. The % decrease in the fluorescence intensity of pyrene was calculated as follows:

1 $${\% }{\text{ Decrease in fluorescence}} = [({\rm I}^\circ {-}{\rm I}^{\rm t} )/{\rm I}^\circ ] \times 100$$

**Fig. 6:**
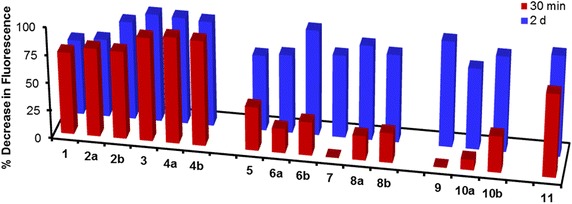
Percent decrease in fluorescence intensity after 30 min and 2 d exposure to the dendrimer films

In this equation, I° is the fluorescence intensity of pyrene at 370 nm in the absence of the branched structure and I^t^ is the fluorescence intensity of pyrene at 370 nm at a specific time point (t) after the film and saturated pyrene solution were combined. Figure 6 compares the percent decrease in pyrene fluorescence intensity at the 30 min and 2 day time points for **1**–**11**. Benzyl ethers **1**–**4b** were shown to be two times more effective in removing pyrene from water relative to **5**–**11**. This trend was true regardless of core type or generation.

The decrease in pyrene signal was dependent on generation size for the benzyl ether architecture. Within 30 min, first generation **3**–**4b** resulted in an average of 94 % reduction of pyrene fluorescence compared to 77 % for zero generation **1**–**2b**. No difference in fluorescence intensity between generations was apparent with cyclohexanes **5**–**8b** and the data could not be determined for the ‘reverse’ or benzyl amine systems since no comparable branched systems could be prepared. Furthermore, core size had minimal impact on pyrene removal (i.e. 92 % decrease in fluorescence intensity with **3** compared with 95 % with **4a**). Exposure of pyrene to benzyl amine **11** was the second efficient system, with a 77 % decrease in fluorescence intensity compared with benzyl ether **2b**.

The fluorescence profile changed when the film was exposed to the pyrene for a longer time period. As seen in Fig. 6, all dendrimeric families demonstrated the ability to effectively remove pyrene from water, with a 67–96 % decrease in fluorescence intensity after 2 days. Generation size or core composition did not greatly influence the ability of the branched system to remove pyrene. Cyclohexanes **5**–**8b** were the least effective, showing only an average 78 % decrease in pyrene fluorescence compared with 85 % for **1**–**4b**, 84 % for **9**–**10b**, and 91 % for **11**. No additional reduction in the pyrene fluorescence intensity was observed after 2 days (Fig. 6).

**Fig. 7:**
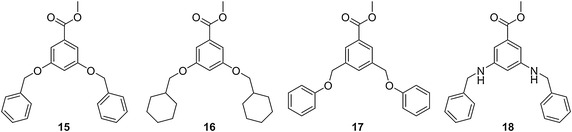
Methyl ester monomers **15**–**18**

**Fig. 8:**
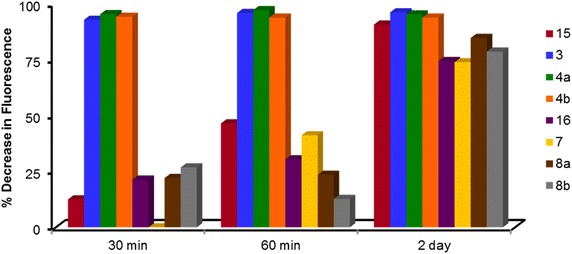
Percent decrease of pyrene fluorescence between **3** and **4b**, **7** and **8b** and corresponding methyl ester monomers **15**–**16**

To determine whether the three armed dendrimer architecture was necessary to cause a decrease in the pyrene signal, fluorescence studies were completed using films of **15**–**18**, the methyl esters of the corresponding monomers of **1**–**11** (Fig. 7). In general, **3**–**4b** and **7**–**8b** were more effective in causing a decrease in the pyrene fluorescence than **15** and **16**, respectively (Fig. 8). The benzyl ether systems provided the largest difference in pyrene fluorescence when comparing **15** (12 % decrease) with **3**–**4b** (average 94 % decrease) at thirty minutes, unlike the cyclohexane systems which exhibited little difference in decreased pyrene fluorescence whether with **7**–**8b** or **16**. The difference in fluorescence intensity between the methyl ester monomers and corresponding branched systems disappeared after 2 days. These results indicate that the dendritic architecture is necessary for effective removal of the pyrene, while the composition of the monomer does impact how effective the dendrimer is in removing the pollutant.

The decrease in pyrene intensity upon exposure to the dendrimeric films suggested an interaction between pyrene and the films. Given the non-polar nature of pyrene and the absence of strong hydrogen bonding donors and acceptors, it was likely that complexation occurred through non-specific hydrophobic interactions. Over the course of 2 days, the film became saturated with pyrene, resulting in its inability to remove additional molecules. Upon removal of the saturated aqueous solutions from the dendrimer films after 2 days, the integrity of the film was analyzed by ^1^H NMR spectroscopy. No degradation of the dendrimer was observed.

Pyrene inclusion formation constants were calculated using the 2 day time points obtained since it was determined that the system reached saturation through fluorescence studies within that time period. A calibration curve was derived which related the pyrene concentration in water to its fluorescence intensity (Monaco et al. 2013). The inclusion formation constant was derived from the following equation:

2 $${\text{Dendrimer }}\left( {s} \right) + {\text{pyrene }}\left( {aq} \right) \to {\text{dendrimer}} - {\text{pyrene complex }}\left( {s} \right)$$

Since the dendrimeric structure existed as a thin solid film, the complex formation constant could be simplified to K = 1/[pyrene]. Inclusion formation constants between 10^9^ and 10^11^ were comparable with other dendrimer and macromolecular solid state systems prepared in a similar manner (Table 1) (Ma and Li 1999; Arkas et al. 2006). The large magnitude of these values suggests a strong interaction between pyrene and **1**–**11**.

**Table 1:** Calculated inclusion formation constants between compounds **1**–**11** and pyrene

Entry	Dendrimer	K (M^−1^)
1	**1**	3.6 × 10^9^
2	**2a**	3.7 × 10^9^
3	**2b**	1.1 × 10^10^
4	**3**	7.9 × 10^10^
5	**4a**	5.2 × 10^10^
6	**4b**	8.2 × 10^10^
7	**5**	7.4 × 10^10^
8	**6a**	2.4 × 10^10^
9	**6b**	1.1 × 10^11^
10	**7**	1.6 × 10^10^
11	**8a**	1.1 × 10^11^
12	**8b**	5.2 × 10^10^
13	**9**	1.7 × 10^11^
14	**10a**	2.7 × 10^10^
15	**10b**	8.0 × 10^10^
16	**11**	2.4 × 10^10^

**Fig. 9:**
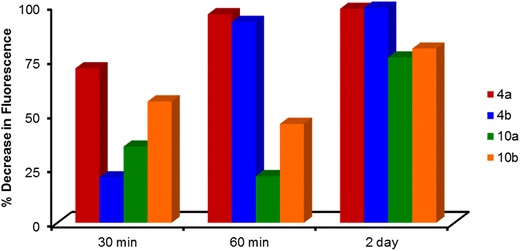
Percent decrease in fluoranthene fluorescence after exposure to benzyl ether and ‘reverse’ benzyl ether structures

Additional studies examined the ability of the dendrimeric families to remove fluoranthene and anthracene. Benzyl ether **4a** and **4b** were more effective in decreasing the fluoranthene fluorescence intensity than **10a** and **10b** after 30 min (Fig. 9). The selectivity eroded at the 2 day time point. Yet, the branched systems were less effective in removing fluoranthene than pyrene as seen by **4a** with a 71 % decrease in fluoranthene fluorescence intensity compared with a 95 % decrease in pyrene fluorescence intensity after 30 min. A similar trend was observed exposing the films with anthracene. Although **4a**–**4b** and **10a**–**10b** removed the PAHs from the aqueous solution, the studies with anthracene and fluoranthene were not as reproducible as pyrene and thus were not continued.

The results described above illustrate that the films effectively removed pyrene from an aqueous solution with some selectivity at the 30 min time point. The benzyl ether family was most effective in removing pyrene from water. The core size did not significantly affect the removal of pyrene from water as dendrimers constructed from the larger cores **13** and **14** gave similar results as dendrimers constructed from the smaller core **12**.

Dendrimeric composition was observed to impact the ability to remove pyrene after 30 min. It was previously predicted that the cyclohexane **5**–**8** would remove pyrene more effectively than **1**–**4** due to the hydrophobic periphery as stated in previous reports (Arkas et al. 2006). However, the cyclohexane branched systems were the least effective in removing pyrene, potentially due to the bulky cyclohexanes which would inhibit favorable intramolecular interactions with the planar PAH. Furthermore, ‘reverse’ benzyl ethers **9**–**10**, which were constitutional isomers of **3**–**4**, were expected to remove pyrene as effectively as the benzyl ether family but were found to be less effective. Benzyl amine **11** removed pyrene reasonably effectively; however, no clear advantage of **11** over the benzyl ether motif was observed. These initial studies suggest pyrene removal is impacted by the type and connectivity of dendrimeric functional groups.

At the 2 day time point, the above selectivity trends eroded, although the benzyl ether systems qualitatively appeared to be the most effective. This kinetic selectivity effect suggested that the benzyl ether family and benzyl amine **11** may contain a more open structure which accommodates faster complex formation with pyrene. This is likely the case for **11**, which was shown to have a greater hydrodynamic volume in solution compared to ether containing systems (Triano et al. 2015). Cyclohexane and ‘reverse’ benzyl ether structures may exist in a more closed conformation, causing complex formation to be slower. The greater steric bulk of the cyclohexane periphery could readily be imagined to give a more closed structure which disfavors complexation with pyrene. The slower binding in the case of **9**–**10** is less obvious and is currently being examined. Reversal of the benzyl ether bond direction must lead to a significantly different solid state conformation relative to **3**–**4**.

The fluorescence based complexation studies described involve branched systems as solid state thin films. Trends in a solution phase system may be different since the mechanism of pyrene removal from the films is not clearly established. The ideal hypothesis would be that the pyrene molecules fit within the interior dendrimer voids (Baars and Meijer 2000). However, it has also been proposed the phenomenon of ‘hydrophobic collapse’ caused dendrimers to adopt a flat or ‘pancake’ shape in the absence of solvent which may lead to stacking of pyrene onto the dendrimer (Fréchet 2002). Other possibilities include the pyrene lodging itself between the individual dendrimers inside the film or the pyrene forming a thin layer on the dendrimer film. A precise understanding is further complicated by the observation that benzyl ether type dendrons exhibit multiple conformations (Gorman and Smith 2000). Whatever mechanism, two conclusions can be drawn: (1) the structure of the dendrimeric framework kinetically impacts the removal of pyrene from water, and (2) the inclusion of hydrophobic groups to the dendrimer periphery, as in the case of **5**–**8**, does not increase the affinity for hydrophobic compounds such as pyrene.

## Conclusions

To test how subtle structural changes on the architecture impact the complexation of small molecule organic pollutants, a series of structurally related dendrimers were studied using fluorescence spectroscopy based complexation studies with pyrene. Members of the benzyl ether family **1**–**4** were most effective in removing pyrene with selectivity within 30 min of exposure to the PAH. Even though their structures were similar to **1**–**4**, cyclohexane, ‘reverse’ benzyl ether, and amine based systems were not as effective at the same time point, but obtained similar pyrene fluorescence signals as **1**–**4** after 2 days. This observation demonstrates that subtle changes in structure lead to different properties, particularly with respect to small molecule complexation. Furthermore, core composition had little influence on pyrene removal when comparing branched systems containing the same monomer. However, by comparing **4b**, **8b**, **10b**, and **11**, simple modifications to the periphery of a known dendrimer scaffold are not the best approach towards fine-tuning dendrimers as previously suggested. Novel dendrimeric scaffolds, such as the benzyl amine system **11** which contain different functionalities within the aromatic architecture, could offer new properties and advantages over known systems.

The results described above provide insight into the connection between the dendrimer structure and its ability to complex a small organic molecule, towards the goal of designing novel dendrimers with optimal properties. Efforts are currently underway to further understand the exact mechanism of complex formation between a dendrimer and a PAH. Furthermore, solid state analysis of dendrimers **1**–**11** and their corresponding monomers has been undertaken to elucidate the complexation properties of dendrimer films. Such insights into the nature of complex formation between a dendrimer and a small molecule guest will have implications beyond the field of environmental remediation, potentially impacting the broader application of dendrimers and branched macromolecules.

## Methods

### Chemicals

Branched systems **1**–**11** were synthesized, characterized, and purified as described elsewhere (Triano et al. 2015). Pyrene (98 % pure) was purchased from Sigma Aldrich and used without further purification.

### Aqueous pyrene solution preparation

A 1 mL aliquot of a 3.7 × 10^−2^ M solution of pyrene in CH_2_Cl_2_ was added to a 250-mL volumetric flask. The solvent was evaporated and distilled water was added to the mark. The flask was sonicated for 10 min and allowed to sit for 24 h prior to use.

### Aqueous anthracene and fluoranthene solution preparation

A 1 mL aliquot of a 4.2 × 10^−2^ M solution of pyrene in EtOAc was added to a 250-mL volumetric flask. The solvent was evaporated and distilled water was added to the mark. The flask was sonicated for 10 min and allowed to sit for 24 h prior to use.

### Dendrimer film preparation

Dendrimer films were created by dissolving 0.02 mmol of the branched system in 5 mL of CH_2_Cl_2_, adding the solution to a 100-mL beaker, and removing the solvent with a stream of air. The films were protected from dust and other contaminants by placing parafilm over the opening.

### Monomer film preparation

Films were created by dissolving 0.06 mmol of the monomer in 5 mL of CH_2_Cl_2_, adding the solution to a 100-mL beaker, and removing the solvent with a stream of air. The films were protected from dust and other contaminants by placing parafilm over the opening.

### Fluorescence spectroscopy studies

Fluorescence data were obtained on a Jobin–Yvon Horiba FluoroMax-3 Spectrofluorimeter (Horiba Scientific, USA) using 325 nm as the excitation wavelength. The fluorescence emission spectrum was obtained from 340 to 500 nm.

*Study without film* To a 100-mL beaker was added 25 mL of filtered saturated pyrene solution. After time points of 30 min, 60 min, and 24 h, an aliquot was removed, filtered through a cotton plug into a cuvette, and its fluorescence measured.

*Study with film* To each beaker containing a film was added 25 mL of the filtered saturated pyrene solution. After time points of 30 min, 60 min, and 24 h, an aliquot was removed, filtered through a cotton plug into a cuvette, and its fluorescence measured. The analysis was performed in duplicate.

As a control, the branched systems were shown to be stable to the conditions of the fluorescence studies. Thin films exposed to deionized water for 24 h were dried, and then ^1^H NMR samples were obtained from the dendrimer films. These samples gave spectra at 300 MHz, using a Bruker AV-300 High Performance Digital NMR Spectrometer (Bruker, USA), which were identical to **1**–**11**, respectively.
